# Supplementary material for: Changes in the Urine Metabolomic Profile in Patients Recovering from Severe COVID-19
Source: Metabolites. 2023 Feb 28;13(3):364. doi: 10.3390/metabo13030364 (PMC10058594; doi:10.3390/metabo13030364)
Supplement: Supplementary file 1 [file metabolites-13-00364-s001.zip › metabolites-2237593-supplementary.pdf]

Supplement S1

Results from discriminatory analysis via cross-validated Random Forest for further combinations of groups, not included in the main manuscript with the most important metabolites responsible for discrimination

**tested system**	**Features ranked by their contributions to classification accuracy**	**discriminatory performance - OOB error**	**AUC value (for binary systems)**
**A-B-C-ctrl**	hippurate, citrate, hypoxanthine, pyruvate, carnitine, alanine, acetate, formate	0.207	-
**A-B-C**	hippurate, citrate, hypoxanthine, carnitine, alanine, acetone, acetate, formate	0.176	-
**A-B**	hypoxanthine, alanine, formate, hippurate, carnitine, citrate	0.196	0.913
**A-C**	hippurate, citrate, alanine, acetone, carnitine, tyrosine, glycine	0.08	0.976
**B- C**	carnitine, citrate, hypoxanthine, acetone, hippurate, formate, alanine	0.09	0.933
